# Early silent coronary bypass graft occlusion following coronary bypass surgery, implication of routine coronary computed tomography angiography

**DOI:** 10.3389/fcvm.2024.1400637

**Published:** 2024-05-23

**Authors:** Islam Salikhanov, Luca Koechlin, Brigitta Gahl, Oliver Reuthebuch, Michael Zellweger, Philip Haaf, Jens Bremerich, Maurice Pradella, Christian Müller, Denis Berdajs

**Affiliations:** ^1^ Department of Cardiac Surgery, University Hospital Basel, Basel, Switzerland; ^2^ Department of Cardiology, University Hospital Basel, Basel, Switzerland; ^3^ Department of Radiology, University Hospital Basel, Basel, Switzerland

**Keywords:** early coronary bypass occlusion, coronary bypass surgery, coronary computed tomography, CABG, coronary angiography

## Abstract

**Objective:**

To evaluate incidence and predictors of early silent bypass occlusion following coronary bypass surgery using cardiac computed tomography angiography.

**Methods:**

A total of 439 consecutive patients with mean age of 66 ± 10 years comprising 17% (*n* = 75) females underwent isolated coronary bypass surgery followed by CT scan before discharge. Graft patency was evaluated in 1,319 anastomoses where 44% (*n* = 580) arterial and 56% (*n* = 739) vein graft anastomosis were performed. Cardiovascular risk factors, demographics, and intraoperative variables were analyzed. We conducted univariable and multivariable logistic regression analyses to analyze variables potentially associated with graft occlusion following CABG. Variables included gender, surgery duration, graft flow, pulsatility index, vein vs. artery graft, and recent MI.

**Results:**

Overall incidence of graft occlusion was 2.4% (31/1,319), and it was diagnosed in 6.6% (29/439) of patients. The difference in occlusion between arterial (2.1%) and vein (2.6%) grafts was not significant, *p* = 0.68. The duration of intervention *p* = 0.034, cross clamp time *p* = 0.024 as well the number of distal anastomosis *p* = 0.034 were significantly higher in occlusion group. The univariate and multivariate logistic regression indicated duration of surgery being predictive for bypass graft occlusion with OR = 1.18; 95% CI: 1.01–1.38; *p* = 0.035.

**Conclusions:**

Early graft occlusion was associated with surgical factors. The number of distant anastamoses, along duration of surgical intervention were, significantly influenced the risk of EGO. Prolonged procedural time reflecting complex coronary pathology and time-consuming revascularization procedure was as well associated to the elevated risk of occlusion.

## 1 Introduction

Coronary artery bypass grafting (CABG) surgery, as a therapy modality, is the standard of care in treatment of severe coronary artery disease (1, 2). It is the most frequently performed cardiosurgical intervention (1, 2). Although CABG, as a procedure, is considered a routine intervention, the potential risk is considerable, especially in patients with advanced cardiovascular risk profile (1, 2).

Perioperative myocardial infarction (MI) is a serious complication following CABG surgery (3, 4). Due to the lack of classical clinical symptoms, the diagnosis of post-operative MI is a clinical challenge and is based on a modification in electrocardiogram (ECG) refractory malignant arrhythmias, an elevation of cardiac biomarkers, and new echocardiography wall motion abnormalities (4, 5). From 25% to 35% of patients with above-mentioned clinical signs suggestive of post-CABG MI have no evidence of coronary bypass graft occlusion (4). Perioperative MI due to the early bypass graft occlusion ranges between 5% and 17% and may have a major impact on early and short-term outcome (6–10). Real prevalence of early bypass occlusion remains unclear due to the absence of symptomatic manifestations of MI in some patients and the consequent lack of adequate diagnosis. Because of a limited number of reports on bypass occlusion in the early postoperative period, early diagnosis of CABG occlusion remains a major unaddressed clinical need (6, 7).

Cardiac CT, which has 96% sensitivity and specificity for diagnosing graft failure, is becoming a feasible, minimally invasive alternative to traditional invasive angiography in the diagnosis of coronary vessel pathology (6–8). This diagnostic tool complements our routine postoperative quality control measures by allowing early identification of graft occlusions before patient discharge. Therefore, this retrospective analysis aimed to determine the incidence and predictors of coronary bypass graft occlusion detected by coronary CT in the early postoperative period. The secondary aim of our study was to investigate the incidence of key postoperative complications, including myocardial infarction, mortality, stroke, and MACCE.

## 2 Material and methods

### 2.1 Ethics statement

The local ethical committee at the University of Basel, Basel, Switzerland (Ethikkomission Nordwest und Zentralschweiz, BASEC-Nr. 2023-01850) approved the protocol of this retrospective study. A written informed consent was waived due to the retrospective nature of the study.

### 2.2 Study population

**Figure 1:**
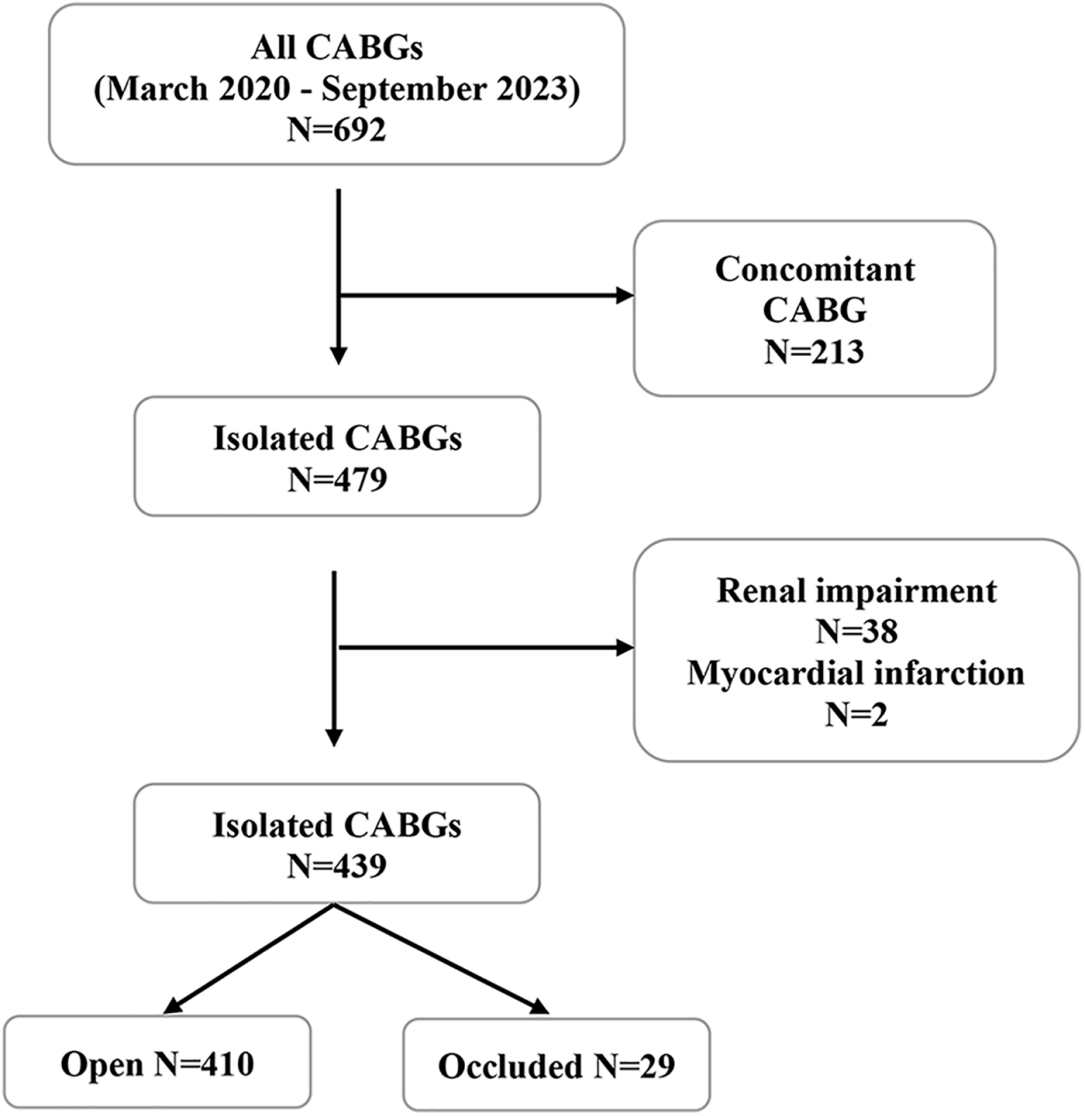
Patient selection flowchart depicting the inclusion criteria for the study on isolated CABG from March 2020 to September 2023. From the initial 690 CABGs, 213 concomitant procedures were excluded. Following exclusion of 38 patients with renal impairment, 439 patients were evaluated, resulting in 410 with patent grafts and 29 with occluded grafts. CABG, coronary artery bypass grafting.

From March 2020 to September 2023, 690 patients underwent coronary bypass surgery (CABG). General exclusion criteria were concomitant surgical procedure, renal failure, refusal of postoperative CT scan, and signs of myocardial infarction (electrocardiographic changes, elevated cardiac enzymes, clinical symptoms of angina pectoris) leading to a postoperative angiographic examination. Renal failure was defined as serum creatinine level >170 mmol/L and estimated glomerular filtration rate (GFR) <30 ml/min/1.73 m^2^, demonstrating at least moderate impairment of renal function (11). We excluded 213 patients due to concomitant procedures and 38 patients due to renal impairment (Figure 1). The remaining 439 patients were classified into two groups. In the first group, we classified those with all anastomoses open, and in the second group, we included those with at least one occluded anastomosis.

### 2.3 Surgical technique

Minimal extracorporeal circulation (MiECC) assisted CABG, or off-pump coronary bypass revascularization (OPCBAG), is the standard procedure for isolated coronary revascularization in our institution. The surgical technique of CABG on MiECC was described earlier (12, 13). In all patients with multi-vessel diseases, CABG was performed through a midline sternotomy. In of-pump, CABG stabilization of the target coronary arteries was accomplished with a tissue stabilizer (Octopus, Medtronic Corporation, Minneapolis, MN). In all cases, an intracoronary shunt (Medtronic Corporation, Minneapolis, MN) for all distal anastomosis was used. At the end of the procedure, just prior to the pericardial closure, mean graft flow (GF), the pulsatility index (PI), and the diastolic filling (DF) were measured. Criteria for acceptable blood flow were as follows: (a) stable shape of blood flow waveform and (b) PI < 5; GF > 15 ml/min. Graft revision was considered in case of insufficient graft flow, low PI conjoined to the new local wall motion in echocardiogram, and/or pathological ST finding in electrocardiogram. Postoperative care included the administration of heparin (14,400 units/h) 6 h post-surgery and aspirin (100 mg) 6 h post-intervention. Clopidogrel (75 mg) or Ticagrelor (90 mg twice a day) was given 6 h post-surgery to all patients with acute coronary syndrome, and all patients were prescribed a lifetime regimen of aspirin (14).

### 2.4 Cardiac computed tomography angiography

Cardiac CT scans, comprising 256 slices, were conducted before patient discharge. Early bypass occlusion was defined as occlusion during the hospital stay. All CT scans were reviewed by both, cardiologists, and radiologists with specific expertise in cardiac CT. We assessed the total number of distal anastomoses, evaluating the number that were patent vs. those that were occluded. Graft occlusion was defined by the lack of contrast medium presence in either (a) the bypass graft or the distal runoff of the anastomosed target vessel, and (b) within the target vessel itself.

### 2.5 Clinical parameters

In-hospital mortality was described as death before discharge. A neurologic event was defined according to the Valve Academic Research Consortium, and a perioperative stroke was expressed as any neurological deficit with or without evidence of cerebral injury in a CT scan and/or in magnetic resonance imaging (MRI) (15). Perioperative myocardial infarction was defined according to the current guidelines (5).

### 2.6 Outcomes

The primary outcome was the incidence of coronary bypass occlusion. Secondary outcomes were incidence of myocardial infarction, mortality, stroke, and incidence of combined major adverse cardiovascular or cerebrovascular events (MACCE). MACCE was specified as a combined event of major in-hospital events such as mortality, stroke, and myocardial infarction.

### 2.7 Statistical analysis

In this study, we aimed to identify variables associated with early graft occlusion following CABG by conducting a comprehensive analysis that included both univariable and multivariable logistic regression models. Variables considered in our analysis were selected based on their potential relevance to graft occlusion, e.g., patient gender, emergency status, duration of surgery, graft flow, pulsatility index (PI), and the time since most recent myocardial infarction (MI). Due to the close association between emergency procedures and recent MI, we opted not to include emergency as a separate variable but instead included a differentiation between vein and artery grafts, acknowledging that graft flow characteristics significantly depend on the graft material. Several intra-operative variables differing between patients with and without occlusion were highly correlated (duration of surgery, CABP-time, x-clamp time, number of anastomoses). So to avoid collinearity, we had to select one of these and chose duration of surgery. The analysis was conducted at the graft level, with patients modelled as a random factor in the model to account for within-patient correlation of multiple grafts. The duration of surgery was operationalized in increments of 10 min.

Statistical evaluations was conducted utilizing Stata 16 statistical software (StataCorp LLC. College Station, TX) and SPSS (IBM Corp. Released 2022. IBM SPSS Statistics for Windows, Version 29.0. Armonk, NY: IBM Corp). For descriptive statistics, the cohort was grouped by outcome into “open” and “occluded” groups. Continuous variables were described as mean ± SD or as median with interquartile range, as appropriate, and compared using Student's *t*-Test or Wilcoxon–Mann–Whitney test accordingly. Nominal and categorical variables were presented as absolute figures and percentages (%). The Mann–Whitney *U*-test facilitated the analysis of numerical data, while binary data were assessed using Pearson's *χ*^2^ test or Fischer's exact test. The analytical framework encompassed preoperative baseline data and perioperative data within the univariate analysis. A statistical significance threshold was set at *p* < 0.05, consistent with conventional standards.

## 3 Results

### 3.1 Patient characteristics

In the final analysis, 439 patients were included. Baseline patient demographics are presented in Table 1. The mean age was 66 ± 10 years, and 17% (*n* = 75) were females. Mean EuroSCORE II was 1.46 [IQR: 0.87–2.72], and 8.9% (*n* = 49) of patients were classified as the New York Heart Association (NYHA) class ≥III/IV heart failure, whereas 47.6% (*n* = 209) of patients were classified as Canadian Cardiovascular Society (CCS) angina class III/IV. Left main disease was present in 15% (*n* = 64) of cases.

**Table 1:** Baseline clinical characteristics.

	Total (*N* = 439)	Open (*N* = 410)	Occluded (*N* = 29)	*p*
Age (years)	66 ± 10	67 ± 10	65 ± 10	0.41
Gender				0.13
Female	75 (17%)	67 (16%)	8 (28%)	
Male	364 (83%)	343 (84%)	21 (72%)	
BMI	27 ± 4.3	27 ± 4.3	27 ± 3.9	0.96
EuroSCORE II	1.46 [0.87–2.72]	1.48 [0.87–2.72]	1.36 [0.95–2.33]	0.85
Angina CCS III or IV	209 (47.6%)	195 (15%)	14 (21%)	0.84
NYHA III or IV	49 (8.9%)	47 (9.0%)	2 (6.9%)	0.98
Diabetes mellitus	179 (41%)	165 (40%)	14 (48%)	0.44
Current smoker	126 (29%)	118 (29%)	8 (28%)	0.58
Hypertension	337 (77%)	316 (77%)	21 (72%)	0.65
Hypercholesterolemia	306 (70%)	282 (69%)	24 (83%)	0.14
COPD	36 (8.2%)	35 (8.5%)	1 (3.4%)	0.50
Previous cardiac surgery	2 (0.46%)	1 (0.24%)	1 (3.4%)	0.13
Extracardiac arteriopathy	95 (22%)	87 (21%)	8 (28%)	0.48
Previous cerebrovascular event	36 (8.2%)	34 (8.3%)	2 (6.9%)	1.00
Left ventricular ejection fraction value (%)	53 ± 11	54 ± 11	51 ± 12	0.15

Values are *n* (%) for categorical variables or mean ± SD for continuous variables. Angina (CCS III or IV) and heart failure (NYHA III or IV) prevalence are shown as counts with the corresponding percentages of the total. Comorbidities are reported as counts with percentages.

BMI, body mass index; CCS, Canadian cardiovascular society; NYHA, the New York heart association; EuroScoreII, European system for cardiac operative risk evaluation; COPD, chronic obstructive pulmonary disease; SD, standard deviation.

Out of 1,319 performed anastomoses, 31 were occluded, and 1,288 were patent. In the occluded cohort, 72% (*n* = 21) of patients were males, and 48% (*n* = 14) received treatment for diabetes mellitus. Cardiovascular risk factors, such as hypertension, impaired renal function, and chronic lung disease, did not differ significantly from patients with no coronary bypass occlusion (Table 1). Patients who underwent CABG with newly diagnosed MI within 7 days before the surgery (35%, *n* = 154) were more likely to have early graft occlusion compared to those with MI more than 7 days before CABG (*p* = 0.045). Elective CABG was performed in 65% (*n* = 286) of cases. The on-pump CABG with MiECC was performed in 81% (*n* = 355) and of-pump in 19% (*n* = 84) cases. Surgical details are presented in Table 2.

**Table 2:** Surgical characteristics.

	Total (*N* = 439)	Open (*N* = 410)	Occluded (*N* = 29)	*p*
Duration of surgery (min)	238 ± 56	236 ± 55	259 ± 54	0.034
CPB-time (min)	107 ± 28	106 ± 28	119 ± 28	0.024
Duration of x-clamp (min)	70 ± 21	69 ± 21	80 ± 18	0.014
Operation urgency				0.52
Elective	284 (65%)	265 (65%)	19 (66%)	
Urgent	155 (35%)	145 (35%)	10 (34%)	
Cardio-pulmonary bypass	355 (81%)	331 (81%)	24 (83%)	1.00
Perfusion				1.00
Off-pump	84 (19%)	79 (19%)	5 (17%)	
MiECC	355 (81%)	331 (81%)	24 (83%)	
Number of distal anastomoses	3.4 ± 1.1	3.4 ± 1.1	3.8 ± 1.1	0.034

Data presented as mean ± SD for continuous variables and *n* (%) for categorical ones. *p*-values assess differences between groups.

CPB, cardiopulmonary bypass; MiECC, minimal invasive extracorporeal circulation.

### 3.2 In hospital outcome

The postoperative results are presented in Table 3. We observed a favorable short-term outcome with no mortality within 30 days post-surgery. The incidence of postoperative complications was relatively low, with stroke occurring in 0.91% of the cases (*n* = 4), and perioperative myocardial infarction being even less frequent, at 0.46% (*n* = 2). The incidence of MACCE during hospital stay was 1.3% (*n* = 6), and 15% (*n* = 68) of patients had atrial fibrillation diagnosed till discharge. The mean duration of ICU stay was 1.8 ± 1.9 days, and the mean hospital stay was 8.8 ± 3.6 days.

**Table 3:** Postoperative outcomes.

	Total (*N* = 439)	Open (*n* = 410)	Occluded (*n* = 29)	*p*
ICU stay (days)	1 [1–2]	1 [1–2]	1 [1–2]	0.6
Definitive pacemaker	3 (0.68%)	3 (0.73%)	0 (0.00%)	1.00
Atrial fibrillation	68 (15%)	63 (15%)	5 (17%)	0.79
Pericardial effusion/tamponade	7 (1.6%)	7 (1.7%)	0 (0.00%)	1.00
Max. CK	595 [410–934]	598 [410–913]	563 [406–1,015]	0.61
Max. CK-MB	37 ± 56	35 ± 55	73 ± 78	0.15
Max. Hs-cTn T	290 [193–595]	289 [192–595]	403 [228–902]	0.14
Pulmonary complication	22 (5.0%)	19 (4.6%)	3 (10%)	0.17
Cerebrovascular event				0.27
TIA	5 (1.1%)	5 (1.2%)	0 (0.00%)	
Stroke	4 (0.91%)	3 (0.73%)	1 (3.4%)	
Length of hospital stay (days)	8.8 ± 3.6	8.6 ± 3.5	10.6 ± 5.1	<0.001

Maximal levels of CK, CK-MB, are reported in units per liter (U/L), and nanograms per liter (ng/L) for Hs-cTn T. Values are mean ± SD for continuous and *n* (%) for categorical variables, with *p*-values for group comparisons.

ICU, intensive care unit; MI, myocardial infarction; CK, creatine kinase; CK-MB, creatine kinase-MB; Hs-cTn T, high-sensitivity troponin T; TIA, transient ischemic attack; SD, standard deviation.

### 3.3 Coronary bypass patency/occlusion and bypass characteristics

CT scan was performed at 6 ± 2 days following CABG. A total of 1,319 distal anastomoses, 43% (*n* = 580) arterial and 56% (*n* = 739) venous, were performed, with a mean of 3.4 ± 1.1 distal anastomoses per patient (Table 2). The overall incidence of bypass graft occlusion was 2.4% (*n* = 31), there was no difference between arterial (2.1%) vs. venous (2.6%) conduits occlusions (*p* = 0.68). A higher number of distal anastomoses was associated with an elevated risk for bypass occlusion (*p* = 0.034) (Table 2). Graft distribution and patency for 439 patients are presented in Supplementary Table S2. Patients with occluded grafts had a longer hospital stay of 10.6 ± 5.1 vs. 8.6 ± 3.5 days (*p* < 0.001). Adverse events, such as atrial fibrillation (*p* = 0.79) and perioperative MI (*p* = 0.13), showed no significant difference between groups (Table 3). Mean duration of intervention was 238 ± 56 min and was significantly longer in patients with bypass occlusion (*p* = 0.034). A similar pattern emerged for cardiopulmonary bypass (CPB) time (*p* = 0.024) and for cross-clamp time (*p* = 0.014).

Mean flow and PI were 50 ± 26 ml/min and 1.6 ± 0.8 in arterial and 65 ± 34 ml/min and 1.5 ± 0.6 in vein grafts, respectively (*p* < 0.01) (Supplementary Table S2). The difference in flow between the open and the occluded vein grafts was significant 67 ± 35 ml/min vs. 51 ± 25 ml/min, (*p* = 0.02), respectively. The mean PI in the occluded graft group was 1.75 ± 0.92, vs. 1.71 ± 0.65 in the open group; *p* = 0.48.

Univariable and multivariable model analysis revealed that only the duration of surgery was a statistically significant predictor of graft occlusion in both, with an odds ratio (OR) of 1.16 (95% CI: 1.00–1.33, *p* = 0.043) in the univariable model and 1.18 (95% CI: 1.01–1.38, *p* = 0.035) in the multivariable model (Table 4). Other variables, including graft flow, PI, graft type (vein vs. artery), and recent MI, were not significant predictors of occlusion in our study. Notably, female gender showed a higher odds ratio (OR = 4.76; 95% CI: 0.72–31.6) in the multivariable model, though this did not reach statistical significance (*p* = 0.106).

**Table 4:** Predictive variables for graft occlusion.

	Odds ratio (95% CI)	*p*
Univariable		
Graft flow	0.99 (0.97–1.02)	0.574
Pulsatility index	1.04 (0.57–1.93)	0.889
Vein graft	1.10 (0.37–3.29)	0.865
MI within 7 days	1.36 (0.30–6.25)	0.693
Duration of surgery	1.16 (1.00–1.33)	0.043
Female sex	4.89 (0.63–38.0)	0.129
Multivariable		0.350
Graft flow	0.99 (0.97–1.02)	0.631
Pulsatility index	1.00 (0.51–1.97)	0.990
Vein graft	1.10 (0.34–3.53)	0.874
MI within 7 days	1.39 (0.28–6.96)	0.691
Duration of surgery	1.18 (1.01–1.38)	0.035
Female sex	4.76 (0.72–31.6)	0.106

The table Illustrates the findings of a univariable and multivariable logistic regression, evaluating potential predictors for graft occlusion. The table lists the Odds Ratios (OR) with their 95% Confidence Intervals (CI), alongside the corresponding *p*-values. The multivariable analysis accounts for potential confounders by including all predictors in the model simultaneously. “MI”, myocardial infarction.

**Figure 2:**
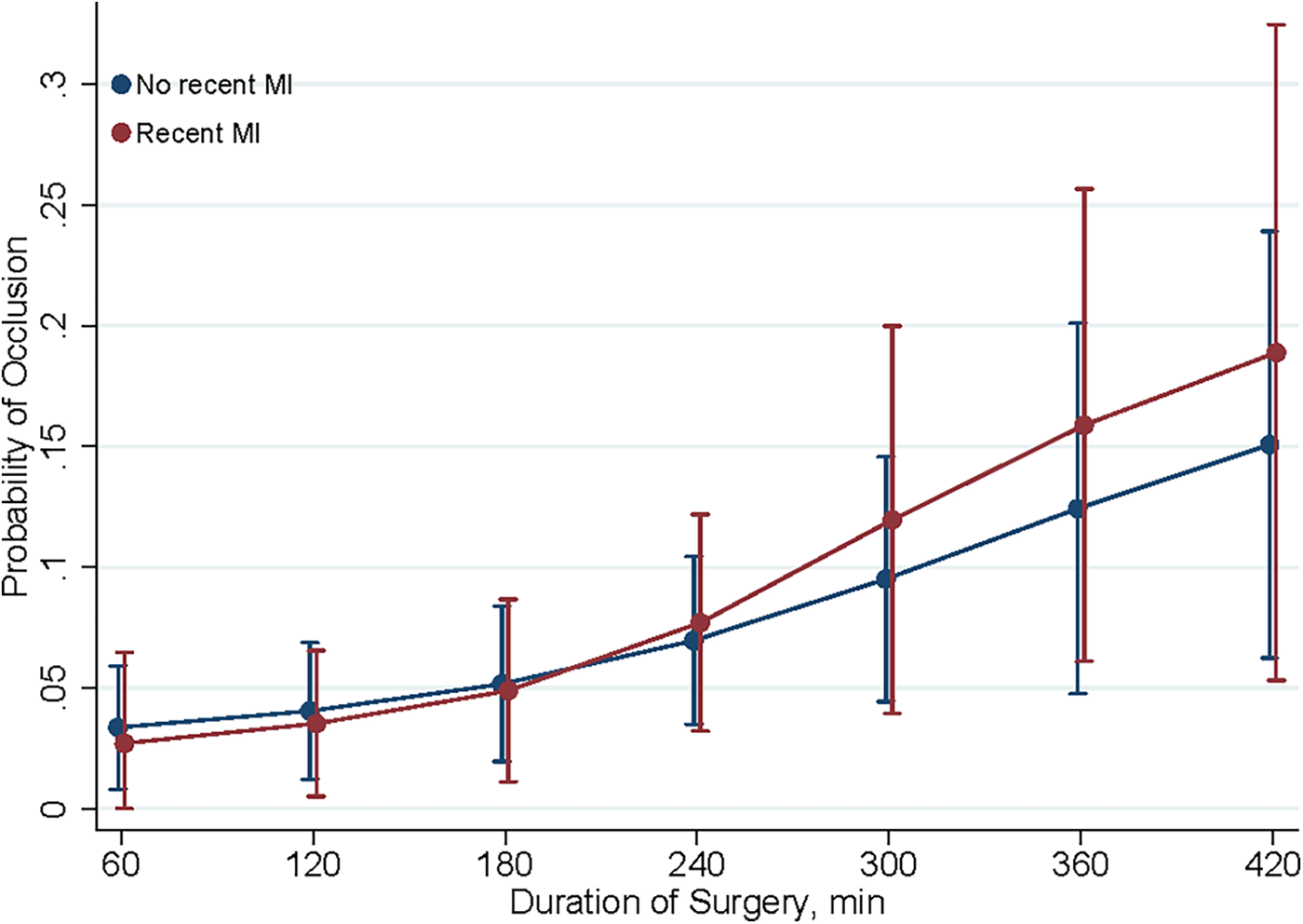
Predictive margins of MI within 7 days for graft occlusion. This figure shows the predictive margins of graft occlusion over different durations of surgery, comparing patients with (red line) and without (blue line) myocardial infarction (MI) within 7 days post-CABG. Vertical bars indicate 95% confidence intervals.

**Figure 3:**
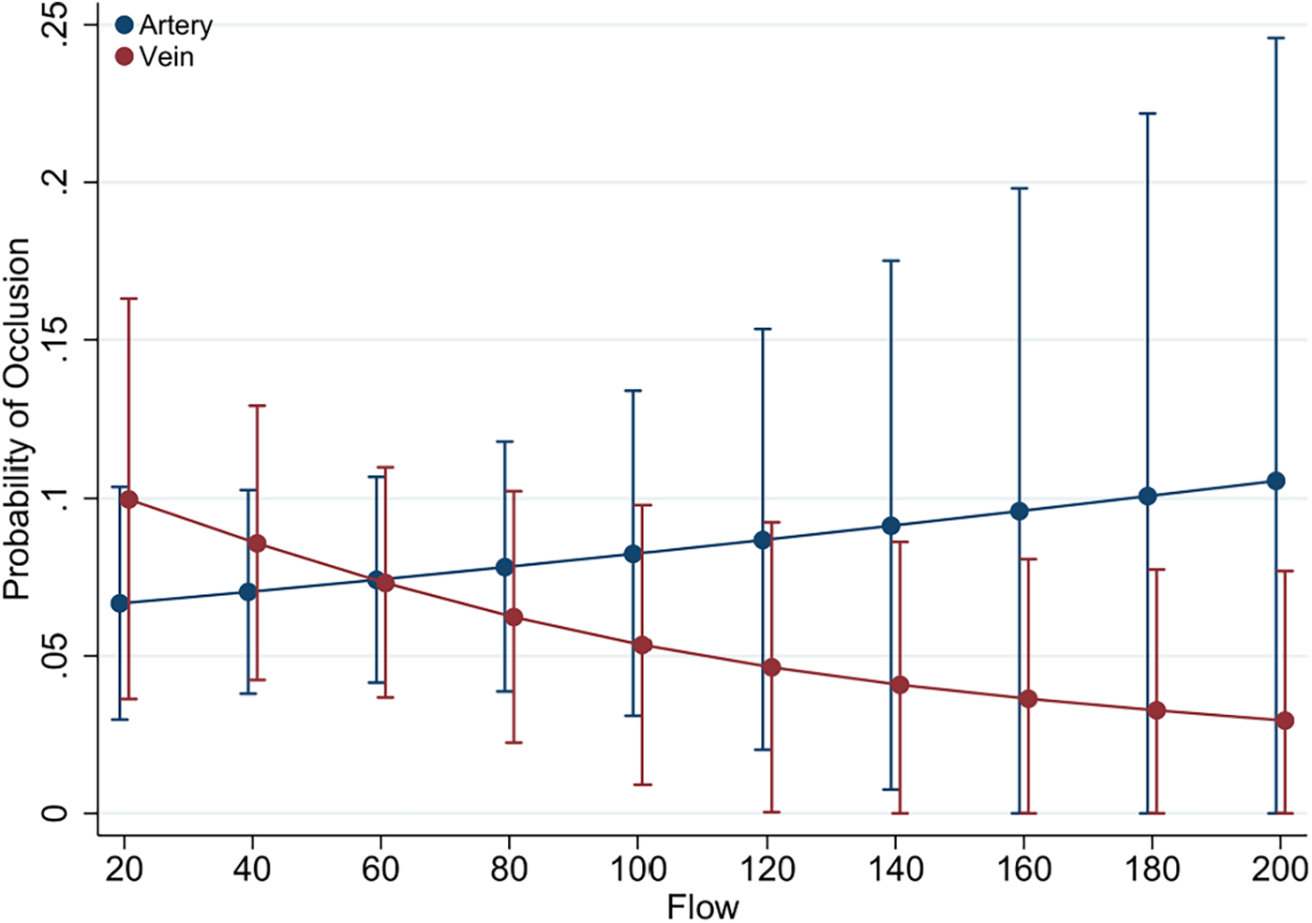
Predictive margins of graft type (vein vs. artery) for graft occlusion. Illustrated here are the predictive margins of graft occlusion across a range of graft flow rates, contrasting venous (red line) with arterial (blue line) grafts. The 95% confidence intervals are represented by the vertical error bars.

The predictive margins for early graft occlusion post-CABG with 95% confidence intervals may be seen in Figures 2–3 present. The influence of MI 7 days prior to CABG on the likelihood of graft occlusion is presented in Figure 2. The patients with a recent MI exhibit a higher predictive margin of graft occlusion compared to those without, and this margin increases with the length of the surgical procedure.

The relationship between graft flow rates and the likelihood of occlusion for different graft types presented in Figure 3. The predictive margins for venous grafts show a slight decrease as flow rates increase, while arterial grafts display a consistent risk across flow rates. The overlapping confidence intervals for both arterial and venous grafts suggest that graft type alone is not a definitive predictor of occlusion risk.

## 4 Discussion

Our study conducted with 439 patients yielded significant findings on graft patency after CABG. The accuracy of post-CABG graft patency is key in the prognosis and long-term outcomes of cardiac surgery patients. We delineated the factors contributing to early graft occlusion by analyzing postoperative CT findings from patients who were operated between March 2020 and September 2023. One of the most important findings was a very low incidence of graft occlusion that occurred in 2.4%, considerably less than reported in recent literature. Recent studies, restricted to a few reports focusing on the mechanism of early coronary bypass occlusion, have reported higher occlusion rates, ranging between 5% and 17% (6, 7). Zientara et al. found an incidence of early graft occlusion of 17% among a cohort of 192 patients, and Han et al. indicated a 5% incidence of early occlusion among a cohort of 346 patients following CABG (6, 7).

We suppose that the low incidence of graft occlusion observed in our study may be attributable to the use of intraluminal shunts serving as a preventive measure against graft failure. Intraluminal shunts serve as temporary conduit ensuring continuous blood flow and are used especially in off-pump and MiECC-assisted CABG procedures in our department. The presence of the shunt in native vessel helps balance the alignment of suture line on the upper edges of the target vessel. We suppose that meticulous surgical technique plays a decisive role in anastomosis integrity and patency, at least in short term. On the other hand, the shunt prevents the accumulation of debris at the anastomosis site, which could otherwise elicit thrombus formation and subsequent graft occlusion (16–18).

In our practice the transit time flow measurement was applied routinely for intraoperative assessment of coronary bypass grafts. The low flow and PI were significantly different in group with graft failure, result consistent with finding in the study by Zientara et al., which reported that the low flow predicted the bypass occlusion (6). However, in multivariate analysis the flow and PI were not predictive for bypass occlusion. This may be in part be probably explained due to the low case load in investigation group. Considering the recent controversy in surgical community on this subject we believe that the role of PI and flow in silent early bypass patency deserves further investigations. Namely the recent guidelines suggest a cut-off value of 5 for an optimal graft, while some surgeons have considered a PI under 3 as an indicator of a good graft, which is in accordance with our results (18, 19).

Given the limited number of patients with graft occlusions (*n* = 29) compared to those without occlusions (*n* = 410), the statistical power is probably insufficient to detect any significant differences in demographic characteristics between the groups. This could explain why, in contrast to existing literature the female gender in our study did not came up as predictor for coronary graft occlusion (6, 7). Although the almost 30% of cohort with bypass occlusion were females in univariate as well in multivariate analysis gender *per se* didn't come up as significant element being associated to the silent coronary bypass occlusion rate. However, the relative high proportion of females in occlusion cohort is remarkable. We suppose that the explanation may be in smaller and more fragile target vessels where consequently the revascularization from technical point of view may be more challenging.

However, the incidence of occlusion in patients undergoing urgent CABG during the index hospital stay was considerably higher. In 35% of cases, CABG was performed as an urgent procedure within the time frame of less than seven days following an acute coronary event. In these, PCI was not performed due to the amenable anatomical conditions, suggesting that complexity of coronary anatomy contributes greatly to surgical failure of anastomoses.

That the underlaying coronary pathology plays an important role in bypass patency is supported by the fact that the duration of surgical intervention was also associated with graft failure, implying that advanced coronary pathology is one of the decisive elements for graft failure. The mean time of intervention was 238 min in our “open” cohort and about 30 min longer in our occlusion group, suggesting a complex coronary situation resulting in more time-consuming revascularization.

Through a retrospective analysis of CT scans out study meticulously examined the incidence of graft occlusion in post-CABG patients. We observed a notably low incidence of early graft occlusion following CABG. Key findings highlighted that a longer duration of surgery was a significant predictor of graft occlusion. Insights of our study are instrumental in influencing clinical decision-making processes, offering a robust foundation for developing targeted interventions. The study underscores the necessity for further clinical research to explore the effects of utilizing intraluminal coronary shunts, aiming to optimize patient outcomes in cardiac surgery.

### 4.1 Limitations

This study was confined to a single-center experience, and as such, the findings may not be generalizable to other settings or populations. The study reflected the practices and patient demographics in one institution and, therefore, may not have captured the full range of surgical techniques, technologies, or post-operative care protocols employed elsewhere.

The impact of early bypass occlusion on clinical outcome, was not completely implemented in our analysis. Regarding the in-hospital outcome there was no difference among two groups. Indeed, the impact of bypass occlusion on follow up outcome here with focus on myocardial ischemia event, revascularization and mortality may be a subject of further investigations. The study's strength lies in its clinical outcomes, underscored by a low incidence of occlusions (31 out of 1,319 anastomoses). However, this very clinical success presents a methodological challenge, as the disparity in group sizes—31 occluded grafts in 29 patients vs. 1,288 open grafts in 410 patients—poses a limitation to the analysis. The smaller sample size of occluded grafts may have decreased the robustness of statistical results; thus, they should be interpreted with caution. Therefore, the marked difference in group sizes may have distorted statistical assessments and complicated the comparison between the groups. While the total size of our sample adds robustness to our study, its single-center nature and the specific patient demographics necessitate further research across multiple centers to broaden the applicability of our findings.

## Data Availability

The raw data supporting the conclusions of this article will be made available by the authors, without undue reservation.
