# Supplementary material for: Early silent coronary bypass graft occlusion following coronary bypass surgery, implication of routine coronary computed tomography angiography
Source: Front Cardiovasc Med. 2024 May 23;11:1400637. doi: 10.3389/fcvm.2024.1400637 (PMC11153815; doi:10.3389/fcvm.2024.1400637)
Supplement: Supplementary file 1 [file Table1.docx]

**Supplementary Table S1.** Preoperative Cardiac History

|  | Total  (N = 439) | Open (N = 410) | Occluded  (N = 29) | p |
| --- | --- | --- | --- | --- |
| History of MI | 238 (54%) | 224 (55%) | 14 (48%) | 0.57 |
| Time since last MI |  |  |  | 0.045 |
| <6 h | 29 (6.6%) | 26 (6.3%) | 3 (10%) |  |
| 6-24 h | 43 (10%) | 43 (10%) | 0 (0.00%) |  |
| 1-7 d | 82 (19%) | 74 (18%) | 8 (28%) |  |
| 8-30 d | 0 (0%) | 0 (0%) | 0 (0%) |  |
| 31-90 d | 0 (0%) | 0 (0%) | 0 (0%) |  |
| >90 d | 0 (0%) | 0 (0%) | 0 (0%) |  |
| Unknown | 15 (3.4%) | 13 (3.2%) | 2 (6.9%) |  |
| Previous PCI | 61 (14%) | 56 (14%) | 5 (17%) | 0.58 |
| Last PCI |  |  |  | 1.00 |
| PCI <24 h | 2 (0.46%) | 2 (0.49%) | 0 (0.00%) |  |
| PCI >24 h; same admission | 3 (0.68%) | 3 (0.73%) | 0 (0.00%) |  |
| PCI >24 h; previous admission | 56 (13%) | 51 (12%) | 5 (17%) |  |
| CAD | 439 |  |  | 0.48 |
| 1 vessel | 13 (3%) | 13 (3%) | 0 (0.00%) |  |
| 2 vessels | 41 (9%) | 40 (10%) | 1 (3%) |  |
| 3 vessels | 321 (73%) | 299 (73%) | 22 (76%) |  |
| Left main disease >50% stenosis | 64 (15%) | 58 (14%) | 6 (21%) |  |

Outlines postoperative outcomes for 439 patients post-CABG, divided into open (N=410) and occluded (N=29) graft groups. Values are mean±SD for continuous and n (%) for categorical variables, with p-values for group comparisons. MI: Myocardial Infarction;

CAD: Coronary Artery Disease; PCI: Percutaneous Coronary Intervention.

**Supplementary Table S2.** Graft distribution and their patency among 439 patients.

| Conduit | Target vessel | Number of patients | Flow (ml/min), mean±SD | PI  Mean±SD | Occlusion |
| --- | --- | --- | --- | --- | --- |
| LIMA | RIVA | 426 | 50±27 | 1.8±0.8 | 1% |
| Vein | CX  OM  Diag  RCA  RIVP  RPLD | 384 | 65±34 | 1.4±0.8 | 5% |
| RIMA | RCA  RIVPO  RCX  RPLD | 79 | 49±27 | 1.8±1.5 | 6% |
| RAD | DM1, DM2  CX | 49 | 41±21 | 1.5± 0.7 | 4% |

Comparison of graft patency across various types of conduits and target vessels. Flow and PI are reported as mean±SD. Patency rates are given in percentages.

The table describes the territories grafted and provides information on graft failures for different conduits, along with other metrics such as flow and pulsatility index (PI). The Left Internal Mammary Artery (LIMA) conduit predominantly grafted to the Right Internal Ventricular Artery (RIVA), with 426 patients. The occlusion rate for LIMA was 1%, indicating a low failure rate in the Left Anterior Descending (LAD) artery. Vein conduits were used to graft multiple target vessels, including Circumflex (CX), Obtuse Marginal (OM), Diagonal (Diag), Right Coronary Artery (RCA), Right Inferior Ventricular Posterior (RIVP), and Right Posterior Left Descending (RPLD), with 384 patients. The vein conduits had a 5% occlusion rate, pointing to potential failures in these non-LAD territories.

The Right Internal Mammary Artery (RIMA) conduit targeted RCA, RIVPO, RCX, and RPLD, with 79 patients. The occlusion rate for RIMA was 6%,. The Radial Artery (RAD) conduit grafted DM1, DM2, and CX, with 49 patients, having a 4% occlusion rate

LIMA: Left Internal Mammary Artery; RIVA: Left anterior descending coronary artery;

CX: Circumflex Artery; OM: Left Marginal Artery; Diag: Diagonal Branch of the Left Anterior Descending Artery; RCA: Right Coronary Artery; RIVP: Right Inferior Ventricular Perforating artery; RPLD: Right Posterolateral Descending artery; RIMA: Right Internal Mammary Artery; RIVPO: Right Inferior Ventricular Posterior artery; RCX: Right Circumflex Artery; RAD: Radial Artery; DM1, DM2: First and Second Diagonal Branches of the Left Anterior Descending Artery; PI: Pulsatility Index; SD: Standard Deviation;
